# Social Media Use and Mental Health: A Global Analysis

**DOI:** 10.3390/epidemiologia3010002

**Published:** 2022-01-11

**Authors:** Osman Ulvi, Ajlina Karamehic-Muratovic, Mahdi Baghbanzadeh, Ateka Bashir, Jacob Smith, Ubydul Haque

**Affiliations:** 1 Department of Public Health & Prevention Science, Baldwin Wallace University, Berea, OH 44017, USA; jsmith16@bw.edu; 2 Department of Sociology and Anthropology, St Louis University, St. Louis, MO 63108, USA; 3 Department of Business Development, Ofogh Kourosh Chain Stores, Tehran 1433894961, Iran; mahdi.baqbanzadeh@gmail.com; 4 Department of Public Health, Amherst College, Amherst, MA 01002, USA; atekaa86@gmail.com; 5 Department of Biostatistics and Epidemiology, University of North Texas Health Science Center, North Texas, Fort Worth, TX 76107, USA; mdubydul.haque@unthsc.edu

**Keywords:** systematic review, social media, mental health, Twitter, Facebook, Instagram

## Abstract

Research indicates that excessive use of social media can be related to depression and anxiety. This study conducted a systematic review of social media and mental health, focusing on Facebook, Twitter, and Instagram. Based on inclusion criteria from the systematic review, a meta-analysis was conducted to explore and summarize studies from the empirical literature on the relationship between social media and mental health. Using PRISMA guidelines on PubMed and Google Scholar, a literature search from January 2010 to June 2020 was conducted to identify studies addressing the relationship between social media sites and mental health. Of the 39 studies identified, 20 were included in the meta-analysis. Results indicate that while social media can create a sense of community for the user, excessive and increased use of social media, particularly among those who are vulnerable, is correlated with depression and other mental health disorders.

## 1. Introduction 

Mental health is defined as emotional, psychological, and social well-being [1]. It plays a role in nearly every aspect of one’s life and can determine how we think, feel, act, respond to stress, relate to others, and even make choices [1]. According to the DSM-5, mental health disorders are “characterized by clinically significant disturbance in an individual’s cognition, emotion regulation, or behavior that reflects a dysfunction in the psychological, biological, or developmental processes underlying mental functioning.” Mental health disorders are common and their etiology ranges from biological factors, such as genes or brain chemistry, to life experiences, such as trauma or a history of abuse [1]. Approximately one in five American adults have some mental health issue, one in ten young people experience a period of major depression, and one in twenty-five Americans report living with a serious mental illness, such as schizophrenia, bipolar disorder, or major depression [1]. 

Furthermore, mental health disorders are influenced by and affect our daily social interactions [1]. Many of our social interactions occur via social media, with individuals spending a significant amount of time on popular social media sites such as Facebook, Twitter, and Instagram, among others. As of December 2019, Facebook reported 2.5 billion monthly active users, Twitter reported 330 million monthly active users, and as of January 2020, Instagram had over 1 billion active monthly users worldwide.

Social media platforms are a great tool for individuals to interact, connect, and support one another [2]. Moreover, many individuals with mental health problems turn to social media platforms to seek support networks and aid others [2]. Social media can further promote a sense of community and assist in keeping relationships not otherwise maintained, which could improve mental health outcomes if correct information and advice are obtained [3]. At the same time, however, increased use of social media may also lead to a constant desire to be connected and can promote negative experiences, which in turn can affect the mental health of the users [3]. Negative effects of increased social media use are especially pronounced for youth; the literature suggests, for instance, that social media use has the potential to amplify the risk of alcohol and drug use among youth [4,5].

In this paper, a meta-analysis was performed to explore the relationship between social media use (Facebook, Instagram, and Twitter) and mental health, using a systematic review of studies from January 2010 to June 2020. The paper additionally assessed the strength of the evidence presented regarding social media and mental health and sought to determine whether a positive effect exists between social media use and mental health.

## 2. Methods 

**Figure 1:**
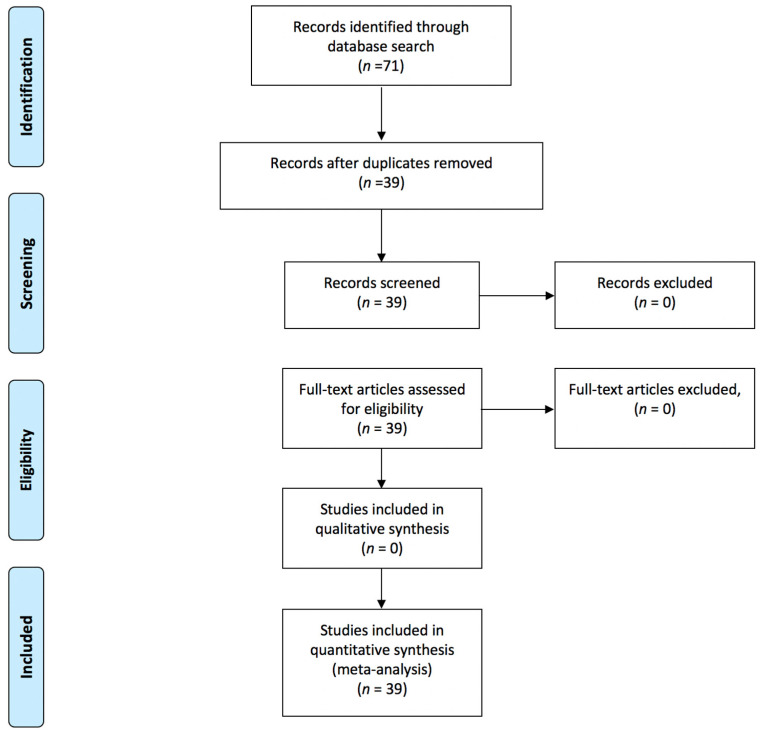
PRISMA 2009 flowchart showing research of records.

A literature search using PRISMA guidelines was conducted to explore the relationship between social media site (Facebook, Instagram, and Twitter) usage and mental health (Figure 1). A multi-database search identified studies published between January 2010 and June 2020. Articles from PubMed and Google Scholar were selected to investigate the relationship of each type of social media site and mental health. While Google Scholar has wide coverage in terms of interdisciplinary scientific studies, it was supplemented and complemented by PubMed due to PubMed’s widely accessible resources and because the database has a provision of MEDLINE and other National Library of Medicine (NLM) resources. Search terms were chosen to broadly capture the various ways social media and mental health have been defined and explored in the existing literature. See Box 1 for a summary of the search strategy and selection process for the systematic review.

Box 1 **Literature search related to social media and mental health.**

- Period search: January 2010 to June 2020.
- Source: PubMed and Google Scholar.
- Search terms: (‘mental health’) AND (‘Social Media’) AND (‘Twitter’ OR ‘Facebook’ OR ‘Instagram’) AND (‘COVID-19′).
- Inclusion criteria: Only articles that displayed use of Social Media, Twitter, Instagram, and Facebook.
- Articles found = 71; Articles included = 39.
- All age groups, male/female, publication date last ten years, all countries and origins.
- Exclusion criteria: Any articles that do not focus on either Twitter, Instagram, or Facebook.
- Articles retained for evaluation = 39.

This study followed Warton et al.’s (2011) recommendation to use GLMMs for the meta-analysis with the logit transformation in meta-analysis problems with single proportions [6]. All the statistical analyses were performed in R studio (version 3.6.1, The R Foundation, Vienna, Austria), using the meta3 and metaphor4 packages [6,7,8,9,10].

### Subgroup Analyses

To identify differences among studies with different scopes, a subgroup analysis with five groups was conducted. Studies were clustered into groups depending on the social media site specified as being the focus of the study. Thus, five groups were created, including only Facebook (labeled F, *n* = 10), only Twitter (labeled T, *n* = 2), only Instagram (labeled I, *n* = 2), all three social media platforms (labeled FTI, *n* = 1), and unknown (social media platform not specified) (labeled U, *n* = 5).

In the subgroup analysis, the sample sizes and the year of publication of the study were also considered (Table S1: Supplementary Materials). For the sample size, we considered the median value of the sample sizes (600) for the cut-off, and for the year of publication studies were categorized as before 2018 and after 2018 (including 2018). To test for the existence of publication bias, we used a funnel plot.

## 3. Results

A total of 71 articles were identified through the Google Scholar and PubMed database search. After duplicates were removed, 39 articles were retained for evaluation. 

Of the 39 studies included, 14 studies focused on Facebook only [3,11,12,13,14,15,16,17,18,19,20,21,22,23].

Two studies focused on both Facebook and Twitter [2,24], and twelve focused on exclusively Twitter [25,26,27,28,29,30,31,32,33,34,35,36].

Three studies included all three social media sites [37,38,39]. Furthermore, three studies focused on Instagram only [40,41,42].

Finally, in five studies, the social media platform included was unknown/not specified [43,44,45,46,47].

The literature spanned vast geographical ranges including Italy [3], Thailand [15], Poland [16], The United States [11,14,17,20,22,28,30,33,38,40,42,43], Germany [18,41], Australia [20,26], Korea [13,29]. The United Kingdom [21,24,35,36], Japan [32], China [44,45], Iraq [39], India [46], and Pakistan [47]. Of the studies included, Twitter was the social media platform most used due to its ease of collecting data by extrapolating large numbers of tweets simultaneously. Some studies were only based on Twitter posts and other studies were based on users of Facebook, Twitter, and Instagram.

The studies included in our meta-analysis employed various analysis methodologies including a study of the association of factors on social media, risk assessment, repeated-measures ANOVA, logistic regression, Poisson multilevel regression, bivariate and multivariate analysis, correlation analysis, advance sentiment analysis, multistage clustering techniques, a sample test of proportional, and statistical inference.

### 3.1. Facebook

Of the 39 included studies, Facebook emerged as one of the main social media sites in 14 of the studies where the relationship between social media and mental health was examined. At least seven of the studies reviewed provided support for a positive relationship between social media use and mental health. For instance, in a survey study conducted in Germany [18], it was found that Facebook users had higher values of certain reported personality traits and positive variables protecting mental health than did non-users. Similarly, while assessing mental health issues such as depression, anxiety, and PTSD, Masedu et al. (2014) reported that Facebook use among adults 25–54 years old had a positive impact on mental health and quality of life outcomes in the years following a disaster [3]. Naslund et al. (2018) found Facebook to be promising for supporting health behavior change among people with serious mental illness [11].

Three of the studies included in the analysis found a negative relationship between social media use and mental health. For example, Hanprathet et al. (2015) illustrated some risks of Facebook usage that affected the mental health status of Thai adolescents in their cross-sectional study [15]. Blachnio et al. (2015) found additional evidence that daily internet use time in minutes, gender, and age were predictive of Facebook intrusion [16]. 

Therefore, studies included in our analysis that focused on Facebook only indicate evidence for both a positive and negative relationship between social media usage and mental health, with slightly more studies evidencing a positive relationship.

### 3.2. Twitter

Of the 12 studies focusing exclusively on Twitter, it was clear that Twitter has been used to raise awareness about many different mental health issues and to help individuals connect and feel that they are not alone [25,26]. For instance, Cavazos-Rehg et al. (2016) reported supportive and knowledge-based awareness tweets about fighting depression to be most common, making up 40% of the tweets reported [27]. Cavazos-Rehg et al. (2016) suggest that health professionals can use Twitter to tailor and target prevention and awareness about mental health [27]. Twitter data have also been found to be useful in providing insight for mental health surveillance before and after traumatic events such as natural disasters [28]. 

Furthermore, Twitter has been useful in the detection and anticipation of mental health issues [28]. For example, Reece et al. (2017) built models to predict the emergence of depression and PTSD by using learning algorithms analyzing the linguistic patterns in Tweets of the sample months before a clinical diagnosis of depression [48]. The results of their study indicated that despite the limitation of 180 characters per tweet, people who were depressed showed signs of depression in their tweets significantly before the actual diagnosis, resulting in the viable option to use Twitter as a predictive depression evaluation tool for clinicians. Similarly, Berry et al. (2017) conducted a study using Text mining methods for Twitter to collect and organize tweets from the hashtag #WhyWeTweetMH [25]. Four overarching themes were derived from the tweets collected: (1) A sense of community; (2) raising awareness and combatting stigma; (3) a safe space for expression; and (4) coping and empowerment [25]. Therefore, evidence from studies focusing on Twitter seems to suggest a positive relationship between social media and mental health.

### 3.3. Instagram

Three of the studies included in the analysis focused on Instagram. Across these studies, the general trend was that Instagram may be a contributing factor in causing body image and self-harm issues in young people. Of the three studies focusing exclusively on Instagram, one study found a relationship between consistent Instagram usage and negative body image and self-harm [40]. This study focused on content posted on Instagram between 18 June 2014, and 30 June 2014, to evaluate the meaning, popularity, and content advisory warnings related to ambiguous non-suicidal self-injury (NSSI) hashtags on Instagram. The sample of 201 Instagram posts led to the identification of 10 ambiguous NSSI hashtags, with some common terms including #selfinjuryy and #MySecretFamily. “#MySecretFamily” was a popular term that described the broader community of NSSI and mental illness. The term #MySecretFamily had approximately 900,000 search results at the time. Content Advisory warnings were only generated by one-third of the relevant hashtags [40]. Another study discussed how image-based social media such as Instagram may become a source of mental health-related information and a tool for health communication [42]. Brown et al. (2019) pointed out in their study how although most of the study participants (80%) had come across expressions of active suicidal thoughts, activity and language use on Instagram did not predict acute suicidality [41]. 

It is important to add that Instagram is the newest platform of the three social media platforms included in this paper, so its lack of history makes it difficult to draw specific conclusions of mental health issues about its long-term use. Nevertheless, based on the inclusion of a limited number of studies, one can conclude that there appears to be a correlation between consistent Instagram usage and the effect on negative body image and self-harm.

### 3.4. Facebook, Twitter, Instagram

Analysis of three studies focusing on all three social media platforms generally indicates that social media use has the potential to influence people’s mental health and psychological well-being. For example, Lis et al. (2015) researched the opinions of psychiatrists on whether social media had adverse effects on psychosis [37]. The study found that 37% of participants believed there was an association between psychopathology and social media sites [37].

In a subsequent study, Lin et al. (2016) assessed depression and social media use across multiple social media platforms in a large and nationally representative sample of young adults [38]. It was found that social media use was significantly associated with increased depression [38]. Most recently, a quantitative survey study by Ahmad et al. (2020) obtained data from the Kurdish social media and found a statistically significant positive correlation between self-reported social media use and the spread of panic related to COVID-19 (R = 0.8701) Results from this study also showed that majority of youth aged 18–35 years are facing psychological anxiety [39]. Therefore, though the number of studies focusing on all three social media platforms included in this analysis is limited, the results of studies included show a negative relationship between social media usage and mental health.

### 3.5. Unknown/Not Specified 

Five of the studies included in the analysis did not specify a social media platform analyzed in their respective study. These studies were more recent in terms of their respective publication date and focus on the relationship between social media use and mental health, primarily during COVID-19. For example, in Hill et al.’s (2019) study, medical students from one US allopathic medical school were asked to complete a 12-item questionnaire [43]. Questions were designed to assess students’ ability to identify, address, and counsel patients on the association between social media and mental health. Results indicated that most of the students believed there could be both a positive and negative effect of social media on mental health [43]. 

Gao et al. (2020) investigated the prevalence of depression, anxiety, and a combination of depression and anxiety (CDA) during the COVID-19 outbreak in Wuhan, China, by using multivariable logistic regression to identify associations between social media exposure with mental health problems after controlling for covariates [44]. They found that more than 80% of participants reported frequent exposure to social media [44]. Findings showed that there was a high prevalence of mental health problems, which were in turn positively associated with frequent social media exposure during the COVID-19 outbreak [44].

Another study conducted by Ni et al. (2020) examined risk factors, including the use of social media, for probable anxiety and depression in the community and among health professionals also in Wuhan, China [45]. A multivariable logistic regression analysis was used to examine these factors [45]. Of the 1577 community-based adults, about one-fifth of respondents reported probable anxiety and depression [45]. Similarly, of the 214 health professionals, about one-fifth of surveyed health professionals reported probable anxiety or depression [45]. Interestingly, social support was associated with less probable anxiety and depression in both health professionals and community-based adults [45]. The results of this study suggest that online platforms can be leveraged to survey community-based adults and health professionals during an epidemic and lockdown [45].

Roy et al. (2020) attempted to assess knowledge, attitude, anxiety experience, and perceived mental healthcare need among the adult Indian population during the COVID-19 pandemic [46]. An online survey was conducted using a semi-structured questionnaire using a non-probability snowball sampling technique [46]. The respondents had a moderate level of knowledge about the COVID-19 infection and adequate knowledge about its preventive aspects [46]. In addition to distress-related social media and sleep difficulties, paranoia about acquiring COVID-19 infection was also reported [46]. The perceived mental healthcare need was seen in more than 80% of participants [46]. The authors suggest that there is a need to intensify the awareness and address the mental health issues of people during this COVID-19 pandemic [46].

In Balkhi et al.’s (2020) study, a structured, self-administered questionnaire was constructed, assessing the psychological impact and behavioral changes about COVID-19 [47]. This research examined data from 400 participants residing in Karachi, Pakistan [47]. The responses were compared based on gender, age, and level of education, to find possible statistical correlations using the chi-square test [47]. The study found increased levels of anxiety due to the use of social media among people below 35 years resulted in avoidance behaviors (*p* = 0.04) [47]. 

In sum, the five studies included in the analysis that did not specify a social media platform suggest not only that the COVID-19 pandemic has exacerbated mental health issues among social media users, but that many have used social media during the pandemic to seek social support for their mental health issues.

## 4. Meta-Analysis Results 

**Figure 2:**
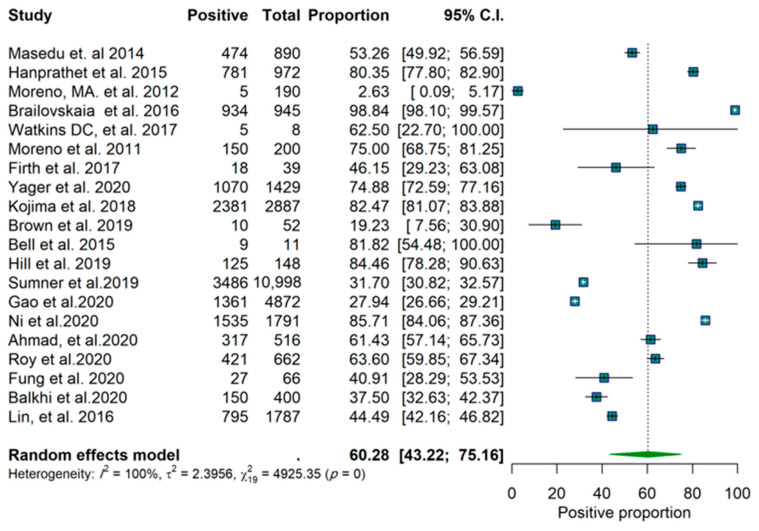
Forest plot of the studies.

**Figure 3:**
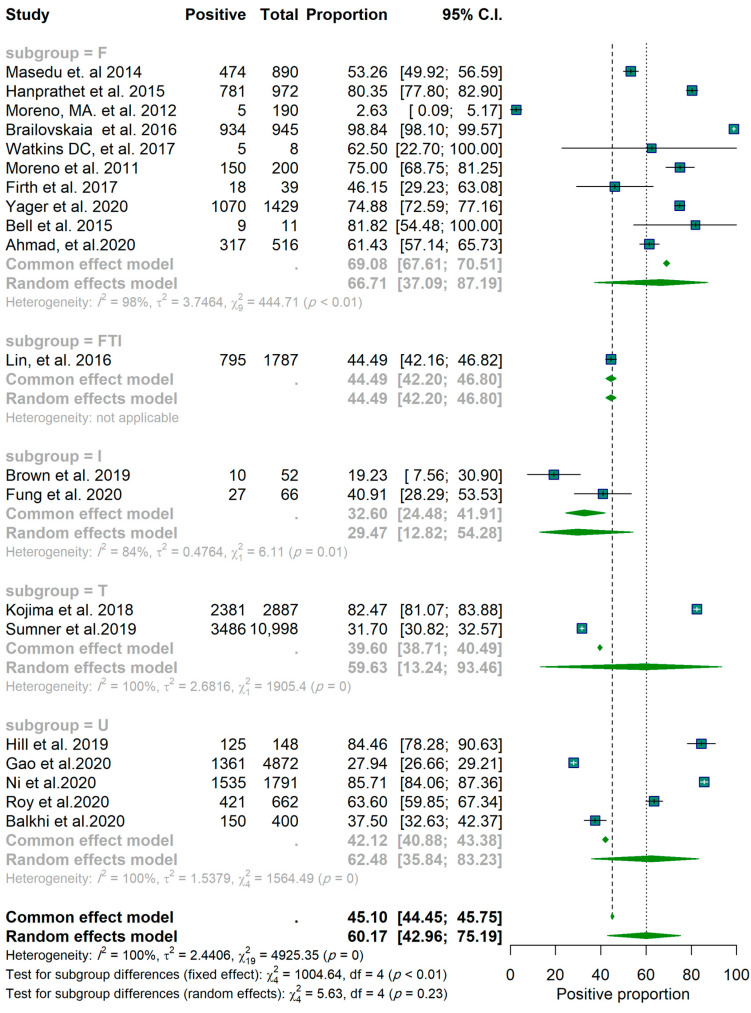
Forest plot of the studies. Grouped by social media platforms.

**Figure 4:**
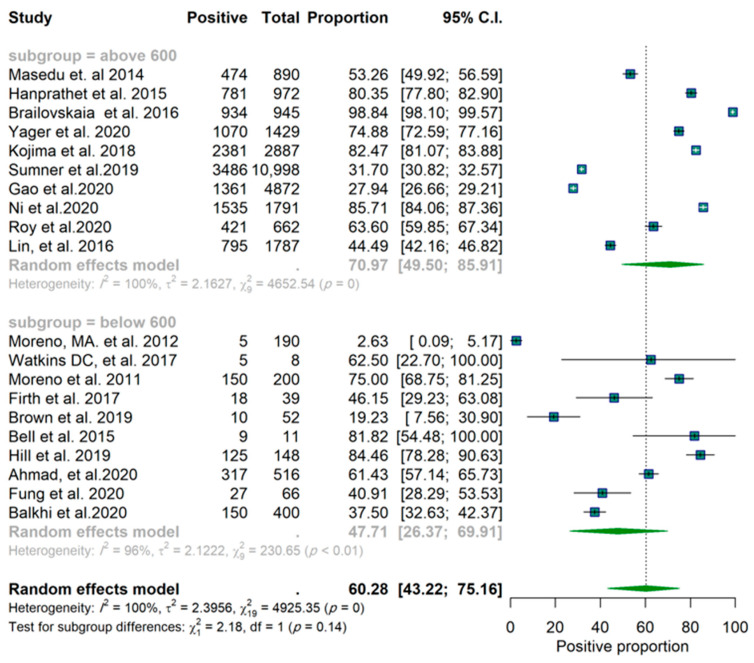
Forest plot of the studies. Grouped by sample size.

**Figure 5:**
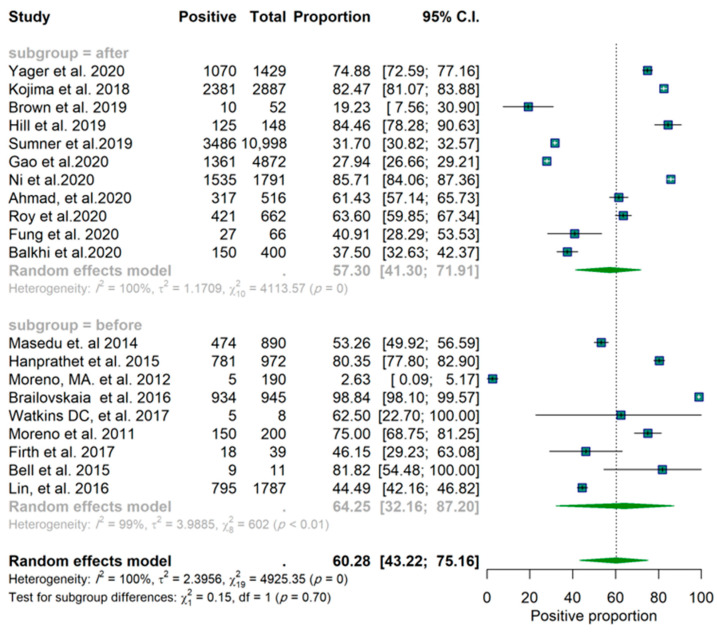
Forest plot of the studies. Grouped by year of publication.

Of the twenty studies, nine reported a proportion lower than 50% for a positive effect (Figure 1). These results are based on the random-effects model. Confidence intervals are based on the Clopper–Pearson interval (exact binomial interval). Here, Q is distributed as a chi-square statistic with k (number of studies) minus 1. It indicates a wide range of values in the outcomes of the studies, and according to I2 = 100%, it was estimated that approximately all of the variance was due to heterogeneity (Figure 1). The forest plots for the studies are found in Figure 2. Considering the scope of the studies (Facebook, Twitter, Instagram, all three, or unknown/not specified), the year of the publication (before 2018 and after 2018), and the sample size of the studies (below 600 and above 600), a subgroup analysis was used to determine the effect of this variation on the pooled results, and the results of these analyses are reported in Table S1, Supplementary Materials. The forest plots for each scenario are illustrated in Figure 3, Figure 4 and Figure 5, respectively. 

**Figure 6:**
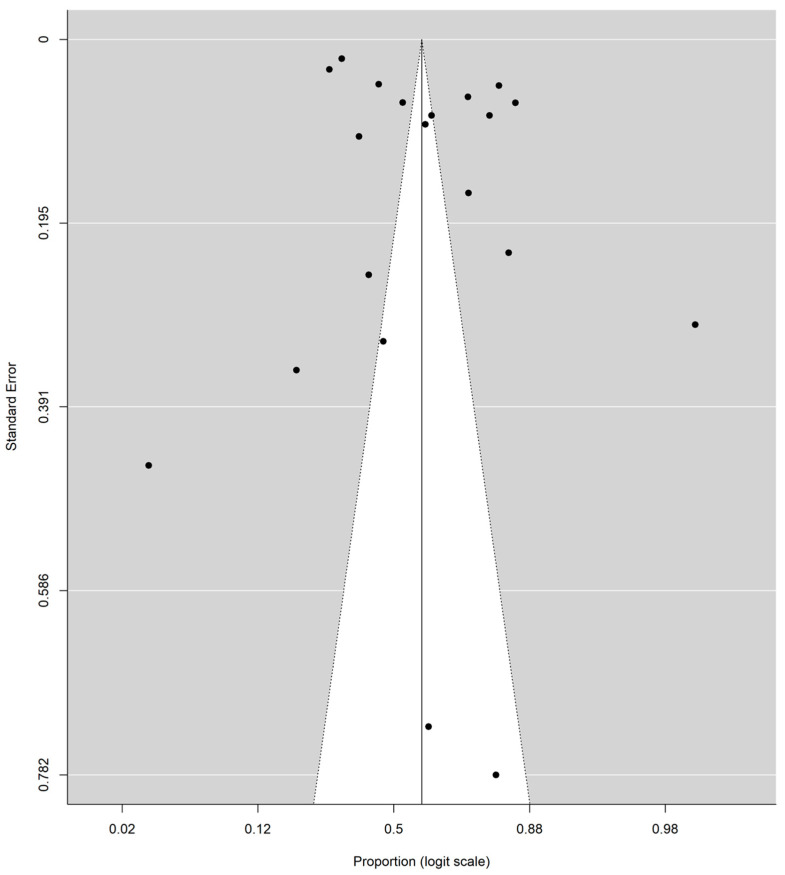
Funnel plot for publication bias.

The subgroup analysis of 10 studies (those which focused only on Facebook) showed an identical pooled proportion of 0.67 (95% CI: 0.38–0.86) with a homogenous characteristic (*p*-value for heterogeneity = 0.09, I2 = 100%). Two studies were sub-grouped based on only Twitter (proportion of 0.59 CI (95% CI: 0.22–0.88)) and two studies focused on Instagram (proportion 0.29 (95% CI: 0.16–0.47)). Among the studies, the studies focused on Instagram both reported a proportion lower than 50% (Table 1). One study was grouped as all three platforms (proportion 0.44 (95% CI: 0.42–0.46)). Five studies were grouped as unknown (proportion 0.62 (95% CI: 0.38–0.81)). According to the groups’ Q (Qb = 7.92, df = 4, *p*-value = 0.09), there was no significant difference found between groups at level α = 0.05 (Table 1). Furthermore, there is no significant difference between studies with sample sizes below and above 600 (Figure 3 and Table 1) and no significant difference between studies before and after 2018 (Figure 4 and Table 1). The funnel plot does not show any clear asymmetrical pattern in publications (Figure 6). 

We also conducted Egger’s regression test. The results of the test (z = −0.19, *p*-value = 0.85) show that there is no significant evidence of publication bias in our study (Table 1).

## 5. Discussion

Social media sites play an important role in individuals’ mental health. In a rapidly evolving world where people experience less face-to-face interaction, understanding the relationship between social media and mental health is essential for the utilization of digital platforms to promote mental health and create a healthier world. The findings of our meta-analysis are mixed and show that social media can both support and hinder one’s mental health. The variations observed depended on the social media platform used as well as whether the study was conducted before or after the COVID-19 pandemic. In general, studies that focused on Twitter and Instagram social media platforms described the worst mental-health expression for the population that was considered in the respective study.

Facebook, the largest and the most used social media site worldwide, connects people from all over the world and enables individuals and communities to easily band together and create movement. Its’ ability for the global exchange of information is unparalleled, as it can bridge people of multiple faiths, nationalities, and orientations in one platform to pursue common goals and raise movements of reform. Facebook can also be used to bridge the worlds of numerous people in a relatively small location through the promotion of health campaigns and community activity to encourage wellness and social interaction.

In terms of mental health, our study shows that Facebook can be and is used to promote mental health through the connection to other users, mental health professionals, and organizations. Our analysis also shows that Facebook can promote mental health among its users by giving them the ability to connect and share their stories with other people who may have the same mental health challenges, making them feel less alone. Using Facebook as a social media awareness platform is an important way to promote mental health through social media. Facebook has “groups” and “pages” that can be used exclusively for mental health awareness. It can also be used to educate individuals and communities about prevention, which could be effective, provided the pages can guarantee anonymity. Facebook’s global reach is quite vast; therefore, any type of mental health intervention employed has the potential of reaching and affecting many individuals. 

Likewise, our study shows that there are mental health risks associated with Facebook overuse. One study that stands out in this finding is by Park et al. (2013), which investigated overall life satisfaction before and after Facebook [13]. Results from this study indicated decreased levels of contentment with the self and life after excessive Facebook usage [13]. Therefore, the relationship between social media usage and mental health when only Facebook is considered varies, and the amount and quality of time spent on Facebook might be an important variable to consider in future studies. 

Twitter is a large platform for people to engage in conversation. It has a strong and loyal audience. Introducing a hashtag and having many people retweet it creates a strong story or interest in the topic it is following. In terms of mental health, Twitter in many ways can serve as a window into users’ mental health. For example, any positive phrases or words related to mental health could be followed by a #mentallove or #mentalhealthlove, and the tweet will be placed in these categories so that any person can search the tweets that are potentially helping others.

Our study indicates that Twitter can be a useful social media platform to combat mental health issues by observing tweets that contain suggestions of depression and then targeting ads or certain pages to respective individuals where they can express their emotions or obtain the necessary help (i.e., nearby medical facilities). Mental health professionals can read and evaluate the tweets to determine if a post shows signs of a mental health issue. People can then be guided to the needed mental health service. With these interventions, professionals can use Twitter to improve the mental health outcomes of many of its users. Policymakers, as well as public health professionals, can use tweets about depression, or other mental health issues, to help find the root cause. They can also reach out to the people who tweet about depression and obtain their feedback on how they can spread awareness.

Since a survey of studies examined in our study suggests a positive relationship between its use and mental health, it is fair to conclude that Twitter may be particularly helpful in promoting an aspect of realness that is fleeting on social media as time goes on. This sense of realness in a virtual community such as Twitter can help minimize skewed mental images, blurring the lines of reality and facade. A true sense of community occurs when role models promote awareness and relate to others as well, so celebrities, policymakers, and athletes tweeting about a mental health issue can also have positive results.

Instagram is a photo-based platform that emphasizes photo and video sharing via its mobile app with over 700 million users worldwide. Our analysis of existing studies focusing on Twitter as a social media platform shows that if Twitter is not used responsibly, it has the potential to negatively influence young people’s body image and self-esteem, such as the evidence from the MacMillan et al.’s (2017) study [49] indicates. 

Though the number of studies focusing on Twitter that are included in our analysis is limited to only three studies, it was clear that young women were the largest group of people that were found to be affected by the negative impact of Instagram, and mostly in terms of their mental health. Matt Kreacher, the author of the #StatusofMind report, suggests that “Instagram draws young women to compare themselves against unrealistic, largely curated, filtered and Photoshopped versions of reality.” All of this is in the palm of their hands for viewing any time of the day or night thus potentially creating a development of body image issues. Because of Instagram and the high level of mental health issues it has been associated with within the literature, the Royal Society of Mental Health proposed social media platforms place a warning on images that have been digitally enhanced or altered photos to reduce feelings of inadequacy [50]. Non-Suicidal Self Injury (NSSI) continues to be a growing and concerning trend on the social media picture-sharing app Instagram, particularly during middle school or early high school years with an estimated prevalence of approximately 7–24% [40].

Other mental health issues that have arisen with the increase in Instagram usage are anxiety, depression, bullying, fear of missing out, and disruptive sleep patterns. Studies have shown that young people who spend more than two hours a day on social media are more likely to report psychological distress [51]. The #StatusofMind report claims that Instagram users may develop a ‘compare and despair’ attitude if they spend too much time on Instagram or other social media platforms.

Other conclusions that can be drawn from our analysis are that studies examined within our parameters that focus on all three social media platforms support the powerful effect that social media has on one’s mental health. Though the number of studies included in our study to arrive at this conclusion is limited, the results of these studies are consistent in showing that increased social media usage equals lower mental health. 

Moreover, the COVID-19 pandemic and social distancing have created an unprecedented setting for examining the relationship between social media usage and mental health. Studies included in our analysis, most of which did not specify a social media platform of focus, inevitably show that while social media usage increased and was rewarding to many users looking for support when the COVID-19 pandemic hit, excessive use also led to mental health issues such as depression and anxiety. Therefore, it can be argued that social media usage during the COVID-19 pandemic specifically is much like a double-edged sword; it can promote mental health, but its overuse can likewise hinder one’s mental health. Mental health consequences of the COVID-19 pandemic will likely be studied well into the future including among ethnic minorities [52,53].

## 6. Implications

Mental health professionals and others promoting psychological health can benefit from learning more about social media and its relationship with mental health. Social media campaigns can likewise promote more knowledge and awareness of specific mental health conditions. A successful advertising campaign can bring awareness to complex mental health issues such as depression and anxiety. Such a campaign has the potential to lead to policymakers flagging or possibly deactivating accounts that promote negative mental health issues. In addition to deactivating negativity, social media sites can promote and advertise positive mental health messages, which would allow the self-help promoting information to reach a wider audience. Using certain hashtags could connect people who are suffering from these issues and give them a needed virtual support group they likely would not have attended in person due to stigma. As an example, the Royal Society for Mental Health is recommending that social media platforms create a “heavy usage” notification to pop up after too much time has been spent online. Social media is not going away, so developing a safe relationship and constructively using social media may not only decrease the negative impact of social media on one’s health but may have a positive impact instead.

## 7. Strengths and Limitations 

A major strength of this review is that it analyzes studies from areas all over the globe, including the United States, the Middle East, Asia, and several European countries. Additionally, the relationship between social media usage and mental health is a particularly important and timely topic to consider. In March of 2020, the WHO declared the COVID-19 outbreak a global pandemic. Global lockdowns required citizens to start spending more time at home, and as a result, social media usage has both increased and changed. More than ever, individuals have turned to social media for socialization, interaction, entertainment, and social support for their mental health.

A limitation of this meta-analysis is the number of databases used to conduct a systematic review, as well as limitations inherent in specifically using Google Scholar and PubMed as databases to identify highly relevant research studies. Each database is limited in its focus and scope, and neither is optimal for topical research. Ideally, multiple databases would provide the optimal and most comprehensive systematic review. Utilizing databases such as PsycINFO, MEDLINE (Ovid), Scopus, CINAHL (Cumulative Index to Nursing and Allied Health Literature), and other educational resources should be considered in future studies. Furthermore, it is well known that most users of social media tend to be adolescents [40], limiting the generalizability of the findings to a wider and older audience. Additionally, only a few of the studies included in the review focused on Instagram only and all three platforms, limiting the conclusions that can be drawn about the relationships between Instagram specifically and mental health.

## 8. Conclusions

Our study shows that individuals suffering from mental health issues use social media as an outlet, and we should continue to use social media to promote wellness. Although these platforms can be a distorted reality for some, they ultimately still serve as platforms where individuals can express themselves. Such expression can be therapeutic for those experiencing mental health issues. Our analysis further shows that Facebook and Twitter have generally been used to both benefit mental health by bringing people of similar mental health situations together and creating a supportive environment. We must continue to strengthen the communities within social networks so that people will be more connected, which will in turn potentially improve their mental health.

The most important finding of this analysis, however, is that there is an untapped potential for early detection using social media platforms. Providing education and tools to navigate social media constructively in schools is a good way to promote self-esteem and mental health. The greatest suggestion to emerge from this study as we move forward into the digital age is to create forums on these social media sites used to benefit the health of the community. Finally, the way people use technology has important implications for healthcare professionals. Social media use should be closely examined from a clinical and public health perspective. 

## Figures and Tables

**Table 1:** Forest plot of the studies. Grouped by social media platforms.

Feature	Group	Number of Studies	Proportion	95% CI	Qb *	*p*-Value
Social Media	Facebook	10	0.6705	(0.3835; 0.8694)	7.92	0.0944
	Twitter	2	0.5963	(0.2287; 0.8803)		
	Instagram	2	0.2964	(0.1664; 0.4705)		
	All	1	0.4449	(0.4220; 0.4680)		
	Unknown	5	0.6252	(0.3849; 0.8164)		
Year	Before 2018	9	0.6425	(0.3216; 0.8720)	0.15	0.6991
	After 2018	11	0.5730	(0.4130; 0.7191)		
Sample Size	Below 600	10	0.4771	(0.2637; 0.6991)	2.18	0.1394
	Above 600	10	0.7097	(0.4950; 0.8591)		

*: Heterogeneity of between groups.

## Data Availability

Data will be shared based upon request through the corresponding author.

## Supplementary Materials

The following are available online at https://www.mdpi.com/article/10.3390/epidemiologia3010002/s1, Table S1: Social media use and mental health: A global analysis.
