# Supplementary material for: Social Media Use and Mental Health: A Global Analysis
Source: Epidemiologia (Basel). 2022 Jan 11;3(1):11–25. doi: 10.3390/epidemiologia3010002 (PMC9620890; doi:10.3390/epidemiologia3010002)
Supplement: Supplementary file 1 [file epidemiologia-03-00002-s001.zip › epidemiologia-1405003-supplementary.pdf]

**Table S1.** Supplementary materials. Social media use and mental health: A global analysis.

| Study Area | Studied by | Sample Size | Social Media | | | Mental Health Findings | | Strength | Weakness |
|---|---|---|---|---|---|---|---|---|---|
| | | | Facebook (N =) | Twitter (N =) | Instagram (N =) | Positive | Negative | | |
| L'Aquila, Italy | Masedu et al., 2014 | 890 | √ | | | 474 | 416 | The first epidemiological study focused on social network use as a factor in mental health. | A cross-sectional design, and no knowledge of the quality of life scores before the study began. |
| Pathumthani, Thailand | Hanprathet et al., 2015 | 972 | √ | | | 781 | 191 | Illustrated some risks of Facebook usage affecting the mental health status of Thai adolescents. | This was a cross-sectional study, so causation between Facebook and mental health issues cannot be determined. |
| Lublin, Poland | Blachnio et al., 2015 | 672 | √ | | | | | Found additional evidence that daily Internet use time in minutes, gender, and age are also predictors of Facebook intrusion. | This study used self-report methods meaning that those who were included filling out the reports themselves without being observed. |
| Madison, Wisconsin | Moreno, MA. et al., 2012 | 190 | √ | | | 5 | 185 | The well-designed study, rigorous data-collection process, validated. | Limited geographical scope, small sample size, studied on older adolescents only. |
| United States | Baker et al., 2016 | 35,044 | √ | | | | | Only included articles that had a quantitative design that compared social network site use and depression. | Limitations in the method of surveying were present in all the studies investigated. |

| | | | | | | | | | |
|---|---|---|---|---|---|---|---|---|---|
| New Hampshire | Naslund, et al., 2018 | 25 | √ | | | | | The study lasted for 6 months, so researchers were able to obtain significant data for all their participants. | A small sample size that lacked racial/ethnic diversity. |
| Bochum, Germany | Brailovskaia et al., 2016 | 945 | √ | | | 934 | 11 | One of the first studies to analyze the relationship between personality traits and mental health in Facebook users and non-users. | Did not consider the participants' time on other social networking sites than Facebook. |
| New South Wales, Australia | Richards et al., 2015 | Literature review | √ | | | | | Identified lack of research in the area of social media and its effect on children and adolescents. | Did not explicitly say how many studies were investigated. |
| Korea, Advanced Institute of Science and Engineerin g | Park et al., 2013 | 55 | √ | | | | | The app, Emotion Diary, successfully evaluated depressive symptoms as well as provided tips and facts to users. | Limited sample size. |
| Michigan, University of Michigan | Watkins DC, et al., 2017 | 8 | √ | | | 5 | 3 | Focused on an ethnic minority | Limited sample size. |
| Many profiles and regions | Moreno et al., 2011 | 200 | √ | | | 150 | 50 | The first study was to identify mental health disclosures on a public social network site. | Only used publicly available profiles, and only one site and university were used. |

| | | | | | | | | | |
|---|---|---|---|---|---|---|---|---|---|
| England, Cambridge University | Naslund JA. et al., 2016 | Commentary | √ | √ | | | | Reaching out to others on social media creates ties and confidence. | Going to social media for support may cause risks and potential rejection. |
| Glasgow, UK | Fergie et al., 2015 | 40 | √ | √ | | | | Used interviews to determine participants' perception of online material regarding diabetes and common mental health disorders. | The sample size is very small to extract data from. |
| Springer Science and Business Media New York | Lis et al., 2015 | 160 | √ | √ | √ | | | The study researched the opinions of psychiatrists on whether social media had adverse effects on psychosis. | Only 48 of the 160 psychiatrists completed the questionnaire so the results are skewed. |
| Worldwide | Berry et. al., 2017 | 132 | | √ | | | | Able to derive themes from their tweet-based study that highlights the "therapeutic benefits of Twitter." | Users may have avoided using their hashtag to prevent being used for research purposes. This created a limitation in the number of tweets the researchers received. Moreover, people were less likely to highlight negative mental health problems to avoid detailing their issues to their followers. |
| California, USA | Ghaznavi et al., 2015 | 300 | | √ | | | | Found that "thinspiration" posts (posts promoting a | The sampling technique resulted in a large percentage of the |

| | | | | | |
|---|---|---|---|---|---|
| | | | | thin figure) purvey social media, so they can be used to target these people who need help. | posts being relatively recently posted. |
| Victoria, Australia | Reavley et al., 2014 | 6358 | √ | The majority of the tweets collected were supportive or neutral of those with depression or schizophrenia. | Inability to determine the proportion of tweets referring to depression and anxiety that were analyzed. |
| New York City during Hurricane Sandy | Gruebner et al., 2017 | 344,957 | √ | Can use Twitter to anticipate acute stress in areas and identify community mental health needs quickly, big sample size. | This is only sampled on a natural disaster. |
| Worldwide | Cavazos-Rehg et al., 2016 | 2000 | √ | Findings can be used to help promote prevention and awareness of depression. | Data lacked contextual meaning since the context was limited by 140 characters in a tweet, leaving out room for explanation. |
| Health related media | Sadah et al., 2016 | 20 million | √ | Gives information as to why and how people use media to learn about health. Further shows how different demographics use different media outlets, large sample sizes. | Some data will lack contextual meaning because of the brevity of Twitter posts and limited information on website searches. |

| | | | | | | | | | |
|---|---|---|---|---|---|---|---|---|---|
| Seattle Children's Research Institute | Moreno et al., 2016 | 200 | | | √ | | | Discovered numerous self-harm hashtags and data about self-harm. | The study was limited to only one form of social media; did not include Twitter or Facebook. |
| University of Mississippi, United States | Firth et al., 2017 | 39 | √ | | | 18 | 21 | Eight-week social media intervention on anxiety in college students examining the impact of dynamic (active) versus static (passive) Facebook content on physical activity behaviors. | The inability to completely blind participants may have limited participant interaction and comfortability in this study. |
| Worldwide | Yager et al., 2020 | 1429 | √ | | | 1070 | 359 | A cross-sectional survey of adult women using an online community sample, with both open and closed questions, was conducted using the survey software Qualtrics. | The cross-sectional nature of the research, and the convenience and snowballing sampling method used. Cannot be sure that the differences in scores observed between those who had, and those who had not seen the film, are attributable to viewing the film. Possible selection bias. |
| Koshu, Japan | Kojima et al., 2018 | 2887 | | √ | | 2381 | 506 | Broadly examined the factors related to PIU among junior-high-school students in Japan. | Since the subject of this study is limited to junior-high-school students in rural areas, caution in generalizing the |

| | | | | | | | | | |
|---|---|---|---|---|---|---|---|---|---|
| | | | | | | | | Clarified relationship of PIU (Problematic Internet use) with sleep and psychological factors. | study’s results is needed. |
| Germany | Brown et al., 2019 | 52 | | | √ | 10 | 42 | The first study to investigate language use on Instagram, one of the most prominent social media platforms among adolescents. | Methodological limitations are related to the exploratory character of this study and the small sample size. Therefore, the results of this study have to be interpreted with caution and cannot be generalized to other populations. |
| The University of Hull, Hull, UK | Bell et al., 2015 | 11 | √ | | | 9 | 2 | The research group studied how people choose to mourn the loss of their dead, and accurately showed how people would respond on Facebook. | The study only showed how people would react on Facebook and neglected other forms of social media. |
| United States | Hill et al., 2019 | 148 | Not applicabl e (n/a) | n/a | n/a | 125 | 23 | Medical students from one US allopathic medical school were asked to take part in a 12- question survey. Questions were designed to assess their ability to identify, | Lack of information regarding demographics of medical students partaking in the study, (e.g., sex, age, year in medical education) |

| | | | | | | | | | |
|---|---|---|---|---|---|---|---|---|---|
| | | | | | | | | address, and counsel patients on the association between social media and mental health. | |
| United States | Sumner et al., 2019 | 10,998 | | √ | | 3486 (31.7%) | 7512 | Broadly assessed dissemination of positive messages on social media. | Limited study period and inability to capture all positive messages. Did not assess how such messages influenced behavior. |
| Wuhan, China | Gao et al., 2020 | 4872 | n/a | n/a | n/a | 1361 | 3511 | Multivariable logistic regressions were used to identify associations between social media exposure with mental health problems after controlling for covariates. | A cross-sectional study among Chinese citizens aged ≥ 18 years old was conducted from 31 January to 2 February 2020. An online survey was used to do a rapid assessment. |
| Wuhan, China Wuhan, China | Ni et al., 2020 | 1791 | n/a | n/a | n/a | 1535 | 256 | A multivariable logistic regression analysis was used to examine factors associated with probable anxiety and probable depression. | Online survey via WeChat, a widely used social media platform in China. |
| Kurdistan, Iraq | Ahmad, et al., 2020 | 516 | √ | √ | √ | 317 | 199 | A quantitative survey methodology to obtain data from Kurdish social media. The | Self-reported data from self-selected participants and the lockdown period was a constraint to gathering more |

| | | | | | | | | | |
|---|---|---|---|---|---|---|---|---|---|
| | | | | | | | | questionnaire was prepared in the Kurdish language. | representative data. |
| India | Roy et al., 2020 | 662 | n/a | n/a | n/a | 421 | 241 | This study attempted to assess the knowledge, attitude, anxiety experience, and perceived mental healthcare need among the adult Indian population during the COVID-19 pandemic. An online survey was conducted using a semi-structured questionnaire using a non-probability snowball sampling technique. | The study is limited to individuals who had smartphones, e-mail IDs, and the ability to speak English. This represents the educated population of the country, so it should not be generalized to the whole population. |
| Literature Search, United States | Fung et al., 2020 | 66 | | | √ | 27 | 39 | Only public health-related, peer-reviewed research articles were included (excluding surveys of self-reported social media use). | No meta-analysis was performed. |
| Worldwide | Abd-Alrazaq et al., 2020 | 160,829 | | √ | | n/a | n/a | Identified the main topics posted by Twitter users related to the COVID-19 pandemic. | The study only analyzed tweets in the English language, which may limit the generalizability of the findings of this |

| | | | | | | | | | |
|---|---|---|---|---|---|---|---|---|---|
| | | | | | | | | | worldwide outbreak. Findings may not represent all the topics discussed by users on Twitter related to COVID-19. |
| Karachi,Pakistan | Balkhi et al., 2020 | 400 | n/a | n/a | n/a | 150 | 250 | A structured, self-administered questionnaire was constructed, based on previously conducted surveys, assessing the psychological impact and behavioral changes about COVID-19. The responses were compared based on gender, age, and level of education, to find possible statistical correlations using the chi-square test. | Being an online survey, bias could not be eliminated, and language barriers could not be bridged. Pre-existing psychiatric conditions among the participants not considered. The study focused on the urban population and the responses in rural areas might have significantly differed. |
| South Korea | Park et al., 2020 | 43,832 | | √ | | n/a | n/a | This study investigates information transmission networks and news-sharing behaviors regarding COVID-19 on Twitter in Korea. | The study’s results may reflect social media users’ views and behaviors during the pandemic rather than the full population’s aggregate opinion. In addition, biases in information-sharing behaviors can exist, as some |

| | | | | | | | | | |
|---|---|---|---|---|---|---|---|---|---|
| | | | | | | | | | users may have produced more content than others. |
| United Kingdom | Ahmed et al., 2020 | 6556 | | √ | | n/a | n/a | This study aimed to develop an understanding of the drivers of COVID-19 conspiracy theories and strategies to deal with such misinformation. This paper performs a social network analysis and content analysis of Twitter. | Search API only retrieved data from public-facing Twitter accounts. Twitter topics are likely to contain automated accounts known as “bots” |
| United States | Lin, et al., 2016 | 1787 | √ | √ | √ | 795 | 992 | This study assessed depression and social media use across multiple social media platforms in a large, nationally representative sample of young adults. | Only used publicly available profiles and only one site and university were used. |
| The University of Hull, Hull, UK | Budhwani et al., 2020 | 193,862 | | √ | | n/a | n/a | A cross-sectional survey of adult women using an online community sample, with both open and closed questions, was conducted using the | Unable to use “gold standard” measures of social media exposure such as ecological momentary assessment or empirical data from social media sites due to the large sample size. |

survey
software
Qualtrics.
